# Mapping the Evidence on Abdominal Weight Training for Respiratory Muscle Strength: A Scoping Review

**DOI:** 10.7759/cureus.104028

**Published:** 2026-02-21

**Authors:** Keita Matsuda, Hiroki Sato, Tomohiro Ikeda, Syo Katayama

**Affiliations:** 1 Department of Physical Therapy, Faculty of Rehabilitation, Kawasaki University of Medical Welfare, Kurashiki, JPN; 2 Department of Rehabilitation Medicine, Okayama University Hospital, Okayama, JPN

**Keywords:** abdominal weight training, breathing exercises, diaphragm, respiratory muscle training, scoping review

## Abstract

This scoping review synthesized evidence on abdominal weight training (AWT), a non-invasive method for respiratory muscle rehabilitation that increases diaphragmatic load, to evaluate its clinical significance. We specifically summarized its effects on respiratory muscle strength, measured by maximal inspiratory pressure (MIP) and maximal expiratory pressure (MEP), diaphragmatic function, and patient outcomes, including ventilator weaning. A systematic search of MEDLINE, CENTRAL, and Web of Science databases conducted on March 13, 2025, identified five studies meeting the inclusion criteria: one randomized trial, two comparative trials, one crossover trial, and one experimental study. Participants included patients with prolonged mechanical ventilation or chronic respiratory failure as well as healthy adults.

The findings indicated that AWT is associated with improved MIP and MEP and enhanced diaphragmatic function in patients with compromised respiratory health. Furthermore, clinical benefits such as improved ventilator weaning rates have been reported, with evidence suggesting that AWT can effectively retrain diaphragmatic breathing patterns. Although AWT appears to be a promising intervention for improving respiratory muscle strength and function in specific populations, the limited number of studies and heterogeneous protocols underscore the need for well-designed trials to standardize training regimens and confirm clinical efficacy.

## Introduction and background

Respiratory muscle training (RMT) has been widely applied across various clinical fields to enhance respiratory muscle strength, exercise tolerance, and functional outcomes in both healthy individuals and patients with a wide range of medical conditions [1,2]. In clinical settings, respiratory muscle function is typically assessed using standardized metrics, specifically maximal inspiratory pressure (MIP) and maximal expiratory pressure (MEP) [1,2]. These indices serve as key clinical outcome measures to quantify the effectiveness of training. In recent years, increasing attention has been directed toward its clinical relevance in patients with respiratory disorders and those requiring prolonged mechanical ventilation, in whom respiratory muscle weakness represents a major barrier to functional recovery and successful ventilator weaning [3]. Accumulating evidence supports the physiological and clinical benefits of RMT. However, its implementation in routine clinical practice remains inconsistent, partly due to a lack of strong and consistent recommendations across international clinical guidance and consensus statements [4,5].

Systematic reviews have highlighted that the clinical application of conventional inspiratory muscle training (IMT) faces significant challenges, including the need for specialized equipment and active patient participation. These requirements often limit its feasibility in critically ill populations, particularly among patients with agitation or delirium [5,6]. Consequently, there is a growing need for accessible, cost-effective alternatives that require minimal equipment and less patient cooperation.

**Figure 1:**
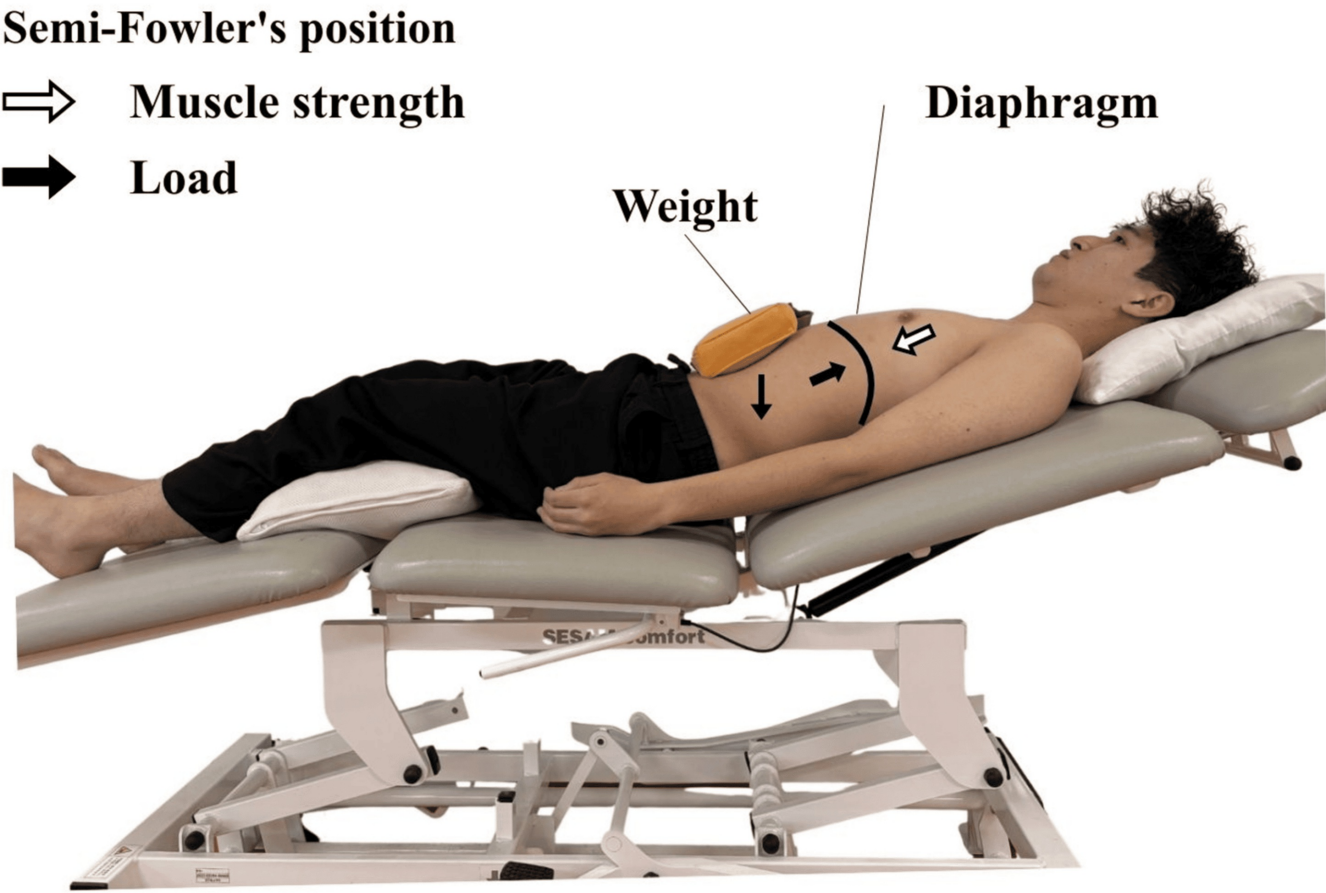
Schematic representation of AWT in the semi-Fowler's position The weight is placed on the supraumbilical region. The white arrow indicates the direction of muscle strength, and the black arrows indicate the load applied by the weight. AWT: Abdominal weight training; Schematic created by authors using Canva (Canva Pty Ltd., Sydney, AUS)

Abdominal weight training (AWT) is a non-invasive method of providing external resistance to diaphragmatic movement by placing a weight on the abdomen during breathing [7]. Specifically, this intervention typically involves the passive placement of a weight (e.g., 1 kg to 3 kg) on the umbilical region of a supine patient (Figure 1). Theoretically, this external load is designed to provide resistance to diaphragmatic contraction, distinct from active weightlifting exercises. Currently, its clinical application has not yet been standardized, and its effects have been investigated in a limited and fragmented manner. While used clinically in certain regions, including Japan, its application has largely developed without internationally standardized protocols or consensus regarding training parameters [8]. Studies examining AWT have been conducted across diverse populations and clinical contexts, and their intervention approaches and outcome measures have not yet been systematically summarized [7,9].

Recent clinical investigations have explored the potential effects of AWT on respiratory muscle strength, diaphragmatic function, and ventilator weaning [7,9]. Yet, the scope and consistency of reported outcomes remain unclear [7,9]. The evidence base is currently fragmented across various study designs and clinical populations. Furthermore, the lack of consensus on protocols and outcome measures creates a significant knowledge gap. Therefore, this scoping review aimed to systematically synthesize available evidence on AWT for respiratory muscle rehabilitation. Our objective was to evaluate its potential clinical significance and inform the design of future high-quality trials.

This article was previously presented as a meeting abstract at the 30th Congress of the Okayama Prefecture Physical Therapy Association on June 29, 2025.

## Review

Materials and methods

Study Design

This scoping review was conducted according to the Joanna Briggs Institute framework [10] and registered prospectively in the Open Science Framework (https://osf.io/fdh78/overview). It was reported in accordance with the Preferred Reporting Items for Systematic Reviews and Meta-Analyses extension for Scoping Reviews (PRISMA-ScR) guidelines, which are open-access and free to use [11]. A completed PRISMA-ScR checklist is provided in Appendix A.

Data Sources and Search Strategies

A search was conducted on MEDLINE, CENTRAL, and Web of Science databases until March 13, 2025. The search terms included “abdominal weight training,” “weight bearing,” and “abdominal pad.” Detailed search terms are listed in Appendix B.

Inclusion and Exclusion Criteria 

**Figure 2:**
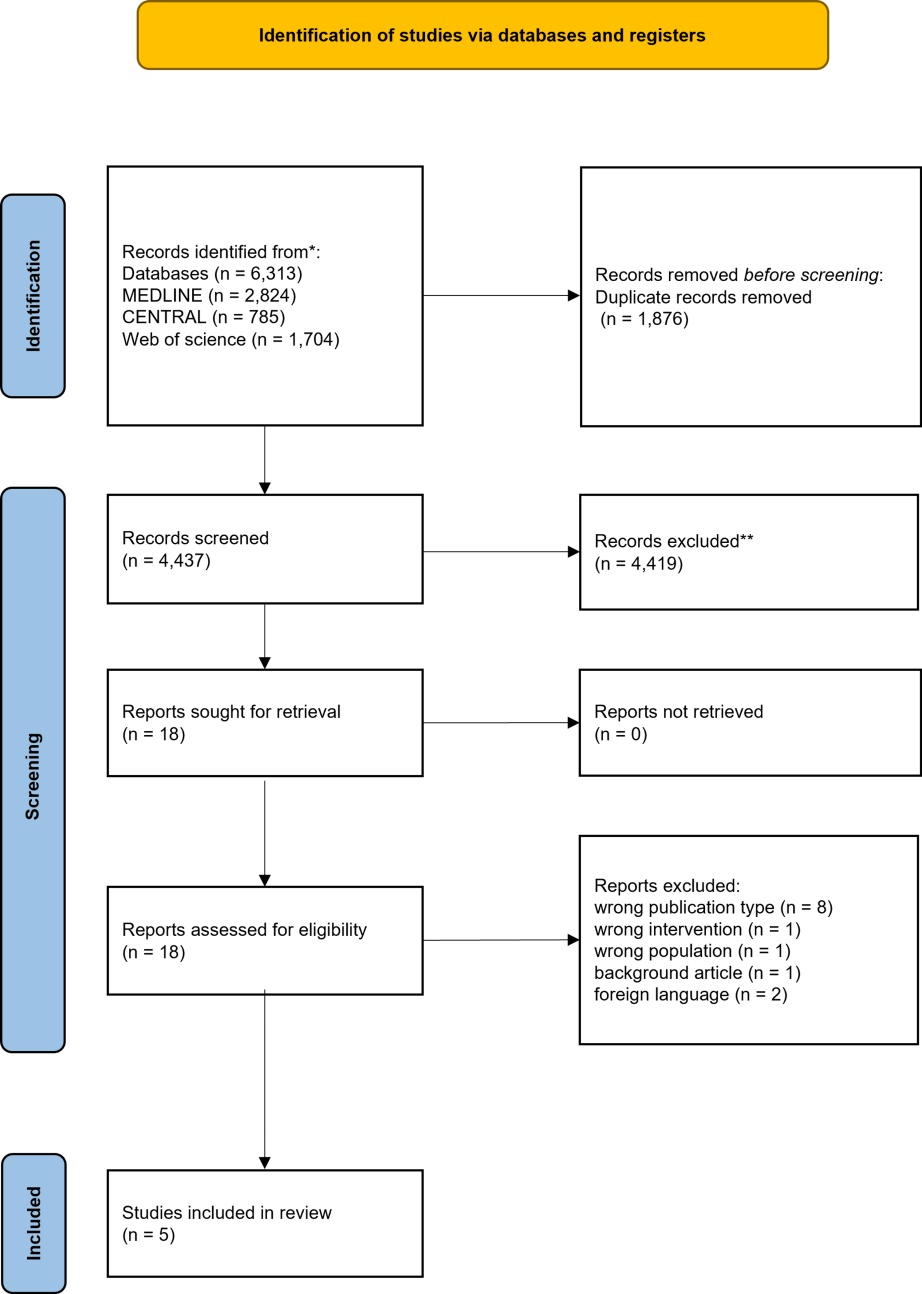
PRISMA flow diagram of the study selection process PRISMA: Preferred Reporting Items for Systematic reviews and Meta-Analyses [11]

Original research studies that investigated the effects of AWT on respiratory function in adults (aged ≥18 years) with no language restrictions were included. Two reviewers independently screened titles and abstracts, retrieved full texts for potentially relevant studies, and resolved disagreements by discussion following a PRISMA flow diagram (Figure 2). We excluded articles that were not original human research, such as reviews, editorials, and animal studies. Conference abstracts and case reports were also excluded to maintain methodological rigor, as these formats often lack essential details regarding the research design, participant characteristics, and intervention protocols. Furthermore, their exclusion helps mitigate potential publication bias, as preliminary findings presented at conferences may not undergo full peer-reviewed publication.

Literature Selection

The initial search yielded 6,313 records. After removing duplicates, 4,437 records were screened. Eighteen articles underwent full-text review, of which 13 were excluded because they did not meet the eligibility criteria. Therefore, five studies were included (Figure 2).

Data Analysis

After paper extraction, the abstracts of the included studies were analyzed for details on authors, year, country, study design, participants, intervention protocols, assessment tools, and outcome measures. Descriptive analysis was performed by summarizing the characteristics of the included studies and synthesizing their findings on the effects of AWT on respiratory muscle strength, diaphragmatic function, and clinical outcomes.

The methodological quality of the included studies was assessed using tools specific to each study design. The Revised Cochrane risk-of-bias tool [12] for randomized trials (RoB 2) was used for randomized controlled trials (RCTs). The risk of bias in non-randomized studies of interventions (ROBINS-I) tool [13] was applied to non-randomized comparative studies. For experimental studies, the Joanna Briggs Institute (JBI) Critical Appraisal Checklist for Quasi-Experimental Studies was used. Furthermore, the JBI Critical Appraisal Checklist for analytical crossover studies was utilized for the crossover trials [14]. Two reviewers independently assessed the risk of bias, and any disagreements were resolved through discussion.

Results

Study Characteristics

The five studies comprised one RCT, two comparative studies, one crossover trial, and one experimental study [7,9,15-17]. The participants varied across the studies: patients with mechanical ventilation (n=40), patients with chronic respiratory failure (n=31), individuals with spinal cord injury (n=9), and healthy adults (n=26). The AWT protocols were notably heterogeneous, with abdominal loads ranging from 1 kg to 23 kg, training frequencies from two to five sessions per week, and durations of up to 12 weeks. Regarding protocol adherence, the study involving patients with mechanical ventilation reported a completion rate of 88.9% overall (40/45), with group-specific rates ranging from 80.0% to 93.3% due to medical complications such as acute infection and abdominal discomfort (Table 1). Methodological quality assessment revealed a generally low to moderate risk of bias; the RCT and experimental study demonstrated low risk, while non-randomized studies presented moderate risks due to inherent design limitations (Appendix C).

**Table 1:** Study characteristics The RSBI is calculated as the ratio of respiratory frequency to tidal volume. RSBI: Rapid shallow breathing index; RCT: Randomized controlled trial; AWT: Abdominal weight training; MIP: Maximal inspiratory pressure; MEP: Maximal expiratory pressure; EMG: Electromyography; NA: Not available

Author, year of publication, country	Study design	Study population	Intervention method	Completion rate	Outcomes		
					Respiratory muscle	Lung function	Others
Hung et al., 2022, Taiwan [7]	RCT	Patients on mechanical ventilation (n = 40, 18 women, mean age 66 years)	AWT group: n = 12, 30 min/day, starting weight 1 kg to 2 kg. Maintain the previous day’s weight and add 0.5kg each time. AWT+ cough machine group: n = 14, besides AWT, perform cough assistance at four to six cycles/session twice daily and five times weekly. Control group: n = 14, two-week time period	AWT group: 80%, AWT + cough machine group: 93.3%, control: 93.3%	MIP, MEP	Respiratory rate, rapid shallow breathing index (RSBI), tidal volume, vital capacity, peak expiratory flow rate, peak cough flow	Status of ventilator weaning achievement and disease severity after intervention
Lin et al., 2024, Taiwan [9]	Comparative study	Patients with chronic respiratory failure (n = 31; 17 women, mean age; 75 years)	Intervention group: n = 17, 2 kg weights, twice daily for three months. Starting at 2 kg, administer five times; if no adverse events occur, increase by 0.5 kg. Control group: n = 14, implemented according to the standard chronic weaning protocol for three months	Intervention group: 100%, control group: 100%	MIP, MEP	RSBI	Diaphragm thickness/motion weaning rate
Monteiro et al., 2012, Argentina [15]	Experimental study	Healthy adults (n = 6, all men, age: 28 to 44 years)	Perform normal breathing and abdominal pattern breathing. For each breathing pattern, sequentially place 1, 2, 4, and 6 kg weights on the umbilical region and measure.	NA	NA	NA	Gastric pressure, transdiaphragmatic pressure/maximal transdiaphragmatic pressure, and tension-time index of the diaphragm
Lin et al., 1999, Taiwan [16]	Comparative study (crossover)	Quadriplegic patients (n = 9, all men, age 20 to 70 years)	Three maximal voluntary breathing maneuvers were performed: an unloaded condition, an abdominal weight load (six repetitions maximum), and an inspiratory resistance load (60% of the MIP). A 30-minute rest was provided between conditions until respiration returned to baseline.	NA	Mouth pressure	Inspiratory flow, inspiratory volume, inspiratory duration	Diaphragmatic electromyography (EMG), sternocleidomastoid EMG, intercostal EMG
Merrick et al., 1981, USA [17]	Comparative study	Healthy adults (n = 30, 15 women, mean age 26 years)	Intervention group: n = 20, 7 kg to 23kg weights, thrice weekly, perform 30 repetitions of maximum voluntary diaphragmatic contractions. Control group: n = 10, live a normal life, six-week period	NA	Peak inspiratory pressure	Inspiratory capacity, peak inspiratory flow	NA

Outcomes and Findings

The results of each study are summarized in Table 2. Two comparative trials reported significant improvements in respiratory muscle strength. An RCT in patients with prolonged mechanical ventilation demonstrated significant gains in MIP following AWT (from 30.5 ± 11.7 to 36.0 ± 10.8 centimeters of water (cmH₂O), p=0.011) and AWT combined with cough machine assistance (from 29.9 ± 12.1 to 36.1 ± 10.4 cmH₂O, p=0.011), whereas no change was observed in the control group. The comparative studies have reported increased MEP. Hung et al. found that MEP significantly improved in the AWT (from 44.40 ± 15.39 to 60.40 ± 19.57 cmH₂O, p=0.033) and AWT+cough machine groups (from 45.86 ± 17.65 to 70.93 ± 20.37 cmH₂O, p<0.001). Similarly, Lin et al. reported a significant increase in MEP in patients with chronic respiratory failure following AWT (from 54.89 ± 24.33 to 66.11 ± 22.36 cmH₂O, p=0.003) [7,9].

**Table 2:** Results and interpretations of the included studies AWT: Abdominal weight training; RCT: Randomized controlled trial; MIP: Maximal inspiratory pressure; MEP: Maximal expiratory pressure

Author, year of publication, and country	Main findings/interpretation
Hung et al., 2022, Taiwan [7]	AWT significantly improved MIP, MEP, and peak cough flow, and significantly reduced the rapid shallow breathing index. The addition of mechanical cough assistance resulted in greater improvements in MEP and peak cough flow. AWT may enhance respiratory muscle strength and cough effectiveness, thereby facilitating ventilator weaning in mechanically ventilated patients.
Lin et al. 2024, Taiwan [9]	AWT significantly improved MIP, MEP, diaphragm thickness, and the rapid shallow breathing index. The intervention group showed a higher ventilator weaning success rate than the control group. AWT may improve diaphragmatic function and respiratory muscle strength, contributing to successful ventilator liberation in patients with chronic respiratory failure.
Monteiro et al., 2012, Argentina [15]	Increasing abdominal loads increased gastric pressure and indices of diaphragmatic activation during breathing. Although diaphragmatic activation increased significantly, the imposed load remained below the threshold required to induce training-related strength adaptations. AWT did not induce respiratory muscle strength gains but may be effective for breathing pattern modulation.
Lin et al., 1999, Taiwan [16]	AWT increased diaphragmatic electromyographic activity, inspiratory flow, and inspiratory volume. Inspiratory resistive loading elicited greater sternocleidomastoid activity and higher negative mouth pressure than AWT. AWT may facilitate diaphragmatic activation, whereas resistive loading may increase accessory muscle recruitment.
Merrick et al., 1981, USA [17]	AWT did not significantly improve peak inspiratory pressure, inspiratory flow rate, or inspiratory capacity. Participants showed increased tolerance to abdominal loading and faster breathing task performance. This AWT protocol may improve task tolerance or endurance but is insufficient to enhance maximal inspiratory strength in healthy adults.

Regarding diaphragmatic function, a prospective trial of patients with chronic respiratory failure showed significantly increased diaphragmatic thickness in the intervention group compared with controls after 12 weeks of progressive abdominal sandbag training (Δ+0.15 ± 0.41 mm vs. −0.38 ± 0.49 mm, p=0.031). Contrastingly, the diaphragmatic excursion did not differ significantly between the groups [9]. An experimental study in healthy adults demonstrated significant increases in transdiaphragmatic pressure (Pdi) and tension-time index of the diaphragm (TTdi). However, the authors concluded that these changes were insufficient to produce effective RMT because the load was only one-third of the load required to induce fatigue in normal participants [15].

For clinical outcomes, AWT was associated with early successful weaning from mechanical ventilation. Hung et al. reported that weaning success rates were higher in the AWT and AWT + cough machine groups (52.9% and 78%, respectively) than in the control group (37.5%). The AWT significantly reduced the RSBI, a key indicator of weaning success [7,9]. Compared with other modalities, a crossover trial demonstrated that AWT and inspiratory resistance loading significantly increased diaphragmatic pressure and electromyographic activity compared with quiet breathing, although no statistically significant difference was found between AWT and inspiratory resistance loading [16]. Furthermore, some investigations reported nonsignificant changes in healthy individuals. Specifically, a comparative study in healthy participants found no significant improvements in MIP, inspiratory capacity, or peak inspiratory flow rate following AWT [17].

Discussion

This scoping review identified five studies investigating the effects of AWT. Collectively, the evidence suggests that AWT may improve respiratory muscle strength, enhance diaphragmatic function, and contribute to favorable clinical outcomes, including successful ventilator weaning. However, the protocols and intervention parameters varied considerably across studies, making it difficult to establish standardized recommendations.

Clinical Implication

The clinical relevance of the AWT is most apparent in populations with compromised respiratory function, such as those with prolonged mechanical ventilation or chronic respiratory failure. In these groups, several studies involving patients with compromised respiratory function reported improvements in respiratory muscle strength following AWT. The documented benefits of AWT, including improved ventilator weaning rates and reductions in RSBI, directly address key challenges in clinical practice [7,9]. In contrast, studies in healthy adults showed that AWT produced only minimal or non-significant changes in respiratory muscle function [15,17]. This divergence highlights the fact that the therapeutic benefit of AWT is most pronounced in individuals with pre-existing respiratory muscle weakness, as the applied load promotes greater contractile effort and functional strengthening of the compromised muscles. This phenomenon may be partly explained by the law of initial values, where individuals with lower baseline function have a greater capacity for improvement compared to healthy individuals who may encounter a physiological ceiling effect. Considering its noninvasive, low-cost nature and minimal equipment requirements, AWT represents a promising and accessible intervention for patients who may not tolerate or have access to more conventional RMT devices.

Scientific Rationale

Underpinning these effects are two primary physiological mechanisms. The diaphragm plays a dual role in human movement, contributing not only to ventilation but also to postural stabilization through the regulation of intra-abdominal pressure [18]. By placing a weight on the abdomen, the intra-abdominal pressure might increase, thereby enhancing resistance against diaphragmatic contraction. Previous physiological studies have shown that external abdominal loading or increased abdominal constraint leads to measurable elevations in intra-abdominal pressure during both resting and active breathing [19]. This external load may compel the diaphragm to generate greater pressure during tidal breathing, thereby strengthening its contractile function over time. Increases in Pdi in response to abdominal loading have been reported, suggesting that the diaphragm is required to generate greater force to overcome externally imposed resistance [15,20]. While increased intra-abdominal pressure is beneficial for diaphragmatic loading, it is important to consider that excessive pressure implies a theoretical risk of discomfort or potential adverse effects, although no such events were explicitly reported in the included studies. Furthermore, increased resistance provided by the abdominal load may promote more deliberate and conscious engagement of the diaphragm, facilitating the acquisition and retraining of diaphragmatic breathing patterns [21,22]. Such resistance-based breathing tasks may function as an intrinsic feedback mechanism, enhancing motor awareness and facilitating relearning of diaphragmatic breathing patterns.

Previous studies have shown that breathing exercises incorporating feedback can modify respiratory behavior and pulmonary function, suggesting that sensory input during breathing practice may contribute to changes in breathing control strategies [23]. Physiological investigations have demonstrated that abdominal loading acutely modifies breathing patterns and increases diaphragmatic electrical activity and transdiaphragmatic pressure [15]. These findings are noteworthy, as they suggest that AWT has the potential to serve as an RMT modality and a form of feedback-assisted breathing practice. While these immediate physiological responses establish a rationale for loading the diaphragm, consistent evidence regarding its long-term training efficacy remains limited.

Limitations of Current Evidence

Several caveats, however, remain. Only one RCT has been conducted, and most available studies are characterized by small sample sizes, thereby restricting the generalizability of their findings. A major limitation across studies is the substantial inconsistency in intervention protocols. This includes variations in load determination, progression strategies, training frequency, and intervention duration. Study populations are also highly heterogeneous, ranging from healthy individuals to critically ill patients requiring mechanical ventilation. The absence of a unified assessment standard for patient selection further limits reproducibility and clinical applicability. Heterogeneous measures, such as MIP and mouth pressure, have been used across studies.

Such variability in protocol design and participant characteristics complicates reproducibility and precludes meaningful evaluation of dose-response relationships. Importantly, these challenges are not unique to AWT; similar methodological limitations, including heterogeneity of training parameters and outcome measures, have been widely reported in studies of inspiratory and RMT [24].

Outcome measures also varied considerably. Marked heterogeneity was observed in the outcome measures used across studies, including respiratory muscle strength, ventilator weaning indices, breathing patterns, and diaphragmatic morphology. For instance, one study used diaphragmatic ultrasonography to assess muscle thickness [9]. While ultrasonography provides valuable data on diaphragmatic morphology, particularly muscle thickness, previous reviews suggest that structural measures alone may not fully reflect functional capacity or contractile performance [25]. Therefore, ultrasonographic findings are most meaningful when interpreted in conjunction with pressure-generating measures. Finally, foundational mechanistic research examining the physiological effects of AWT in healthy populations prior to broad clinical application is limited, highlighting an important gap in the existing literature.

Future Directions

Future research should prioritize the development of standardized protocols for load setting and training frequency, informed by physiological principles, to facilitate comparison across studies. Second, patient populations that are most likely to respond to AWT must be identified to clarify which clinical conditions or disease groups are appropriate candidates for this intervention. Finally, future studies should use comprehensive outcome measures that include traditional pulmonary function tests and advanced assessments, such as diaphragmatic ultrasound (e.g., thickening fraction, excursion) and EMG, to fully evaluate respiratory muscle adaptations.

## Conclusions

This scoping review suggests that AWT may improve respiratory muscle strength and diaphragmatic function, with potential benefits for clinical outcomes, such as ventilator weaning. However, definitive conclusions cannot be drawn because of the limited number of studies, inconsistencies in intervention protocols, methodological variability, and marked differences in study populations. Future well-designed studies are required to establish standardized and effective AWT protocols.

## Appendices

Appendix A

Table 3 features the complete PRISMA-ScR checklist used for this scoping review.

**Table 3:** PRISMA-ScR checklist PRISMA-ScrR: Preferred Reporting Items for Systematic Reviews and Meta-Analyses extension for Scoping Reviews [11]

Section	Item no.	PRISMA-ScR checklist item	Page no.
Title			
Title	1	Identify the report as a scoping review.	1
Abstract			
Structured summary	2	Provide a structured summary that includes (as applicable): background, objectives, eligibility criteria, sources of evidence, charting methods, results, and conclusions that relate to the review questions and objectives.	2
Introduction			
Rationale	3	Describe the rationale for the review in the context of what is already known. Explain why the review questions/objectives lend themselves to a scoping review approach.	3-4
Objectives	4	Provide an explicit statement of the questions and objectives being addressed with reference to their key elements (e.g., population or participants, concepts, and context) or other relevant key elements used to conceptualize the review questions and/or objectives.	3-4
Methods			
Protocol and registration	5	Indicate whether a review protocol exists; state if and where it can be accessed (e.g., a web address); and if available, provide registration information, including the registration number.	4
Eligibility criteria	6	Specify characteristics of the sources of evidence used as eligibility criteria (e.g., years considered, language, and publication status), and provide a rationale.	4
Information sources*	7	Describe all information sources in the search (e.g., databases with dates of coverage and contact with authors to identify additional sources), as well as the date the most recent search was executed.	4
Search	8	Present the full electronic search strategy for at least 1 database, including any limits used, such that it could be repeated.	4 Appendix
Selection of sources of evidence	9	State the process for selecting sources of evidence (i.e., screening and eligibility) included in the scoping review.	5
Data charting process	10	Describe the methods of charting data from the included sources of evidence (e.g., calibrated forms or forms that have been tested by the team before their use, and whether data charting was done independently or in duplicate) and any processes for obtaining and confirming data from investigators.	5
Data items	11	List and define all variables for which data were sought and any assumptions and simplifications made.	5
Critical appraisal of individual sources of evidences	12	If done, provide a rationale for conducting a critical appraisal of included sources of evidence; describe the methods used and how this information was used in any data synthesis (if appropriate).	Not applicable
Synthesis of results	13	Describe the methods of handling and summarizing the data that were charted.	5
Results			
Selection of sources of evidence	14	Give numbers of sources of evidence screened, assessed for eligibility, and included in the review, with reasons for exclusions at each stage, ideally using a flow diagram.	5, Fig. 2
Characteristics of sources of evidence	15	For each source of evidence, present characteristics for which data were charted and provide the citations.	5, Table 1
Critical appraisal within sources of evidence	16	If done, present data on critical appraisal of included sources of evidence (see item 12).	Not applicable
Results of individual sources of evidence	17	For each included source of evidence, present the relevant data that were charted that relate to the review questions and objectives.	5-6, Table 2
Synthesis of results	18	Summarize and/or present the charting results as they relate to the review questions and objectives.	6-7
Discussion			
Summary of evidence	19	Summarize the main results (including an overview of concepts, themes, and types of evidence available), link to the review questions and objectives, and consider the relevance to key groups.	7-8
Limitations	20	Discuss the limitations of the scoping review process.	8
Conclusions	21	Provide a general interpretation of the results with respect to the review questions and objectives, as well as potential implications and/or next steps.	8
Funding			
Funding	22	Describe sources of funding for the included sources of evidence, as well as sources of funding for the scoping review. Describe the role of the funders of the scoping review.	9

Appendix B

Table 4 includes the detailed search terms used to conduct this scoping review.

**Table 4:** Search terms

Database	#	Search query	Results
MEDLINE	#1	((((((((((((((rehabilitation[MeSH Terms]) OR (exercises movement techniques[MeSH Terms])) OR (respiratory therapy[MeSH Terms])) OR (respiratory therapies[MeSH Terms])) OR (agents, respiratory system[MeSH Terms])) OR ("rehabilitation"[Title/Abstract])) OR ("exercise movement techniques"[Title/Abstract])) OR ("respiratory"[Title/Abstract])) OR ("agents, respiratory system"[Title/Abstract])) OR (Respiratory Muscles[MeSH Terms])) OR (Diaphragm[Title/Abstract])) OR (Inspiratory muscle[Title/Abstract])) OR (IMT[Title/Abstract])) OR (Expiratory muscle[Title/Abstract])) OR (EMT[Title/Abstract])	1,385,673
	#2	((("abdominal pad"[Title/Abstract]) OR ("abdominal weight"[Title/Abstract])) OR ("weight bearing"[Title/Abstract])) OR ("abdominal sandbag"[Title/Abstract])	19,268
	#3	animals [mh] NOT humans [mh]	5,315,757
	#4	#1 AND #2 NOT #3	2,824
CENTRAL	#1	(((((((((((((([mh rehabilitation]) OR ([mh "exercises movement techniques"])) OR ([mh "respiratory therapy"])) OR ([mh "respiratory therapies"])) OR ([mh "agents, respiratory system"])) OR (rehabilitation:ti,ab)) OR ("exercise movement techniques":ti,ab)) OR (respiratory:ti,ab)) OR ("agents, respiratory system":ti,ab)) OR ([mh "Respiratory Muscles"])) OR (Diaphragm:ti,ab)) OR ("Inspiratory muscle":ti,ab)) OR (IMT:ti,ab)) OR ("Expiratory muscle":ti,ab)) OR (EMT:ti,ab)	194632
	#2	((("abdominal pad":ti,ab) OR ("abdominal weight":ti,ab)) OR ("weight bearing":ti,ab)) OR ("abdominal sandbag":ti,ab)	3032
	#3	#1 AND #2	785
Web of Science	#1	((((((((((((((ALL=rehabilitation) OR (ALL="exercises movement techniques")) OR (ALL="respiratory therapy")) OR (ALL="respiratory therapies")) OR (ALL="agents, respiratory system")) OR ((TI=rehabilitation OR AB=rehabilitation))) OR ((TI="exercise movement techniques" OR AB="exercise movement techniques"))) OR ((TI=respiratory OR AB=respiratory))) OR ((TI="agents, respiratory system" OR AB="agents, respiratory system"))) OR (ALL="Respiratory Muscles")) OR ((TI=Diaphragm OR AB=Diaphragm))) OR ((TI="Inspiratory muscle" OR AB="Inspiratory muscle"))) OR ((TI=IMT OR AB=IMT))) OR ((TI="Expiratory muscle" OR AB="Expiratory muscle"))) OR ((TI=EMT OR AB=EMT))	1023210
	#2	((((TI="abdominal pad" OR AB="abdominal pad")) OR ((TI="abdominal weight" OR AB="abdominal weight"))) OR ((TI="weight bearing" OR AB="weight bearing"))) OR ((TI="abdominal sandbag" OR AB="abdominal sandbag"))	12114
	#3	#1 AND #2	1704

Appendix C

Table 5 summarizes the low to moderate risk of bias found in the included studies.

**Table 5:** Risk of bias in included studies JBI: Joanna Briggs Institute; MEP: Maximal expiratory pressure; MIP: Maximal inspiratory pressure; PCF: Peak cough flow; RCT: Randomized controlled trial; RoB: Risk of bias; ROBINS-I: Risk of bias in non-randomized studies of interventions; TTdi: Tension-time index of the diaphragm

Author and year of publication	Study design	Appraisal tool	Overall risk	Main bias risks and remarks
Hung et al., 2022 [7]	RCT	RoB 2	Some concerns	Retrospective trial registration; potential measurement bias due to lack of assessor blinding for effort-dependent outcomes (MIP, MEP, PCF)
Lin et al., 2024 [9]	Comparative study	ROBINS-I	Moderate	Lack of statistical adjustment for baseline imbalance in tidal volume; unclear blinding of outcome assessors
Monteiro et al., 2012 [15]	Experimental study	JBI quasi-experimental	Moderate	Small sample size (n=6) and lack of an independent control group. Strength: High physiological measurement reliability (transdiaphragmatic pressure, TTdi)
Lin et al., 1999 [16]	Comparative crossover	JBI crossover	Moderate	Small sample size (n=9) and lack of blinding for participants/assessors. Strength: Randomized sequence assignment and clear 30-minute washout period
Merrick et al., 1981 [17]	Comparative study	JBI quasi-experimental	Low	Presence of an independent control group (n=10); high methodological quality with sophisticated physiological measurement techniques minimizing artifacts
